# Distance-based classifiers as potential diagnostic and prediction tools for human diseases

**DOI:** 10.1186/1471-2164-15-S12-S10

**Published:** 2014-12-19

**Authors:** Boris Veytsman, Lei Wang, Tiange Cui, Sergey Bruskin, Ancha Baranova

**Affiliations:** 1 School of Systems Biology, George Mason University, David King Hall, MSN 3E1, Fairfax, VA, 22030, USA; 2 Computational Materials Science Center, George Mason University, Research I, MS 6A12, Fairfax, VA, 22030, USA; 3 Vavilov Institute of General Genetics RAS, Gubkina str. 1, Moscow, 119333, Russia; 4 Research Centre for Medical Genetics RAMS, Moskvorechye 1, Moscow, 115478, Russia; 5 Moscow Institute of Physics and Technology, Institutsky 9, Dolgoprudny 141700, Russia

## Abstract

Typically, gene expression biomarkers are being discovered in course of high-throughput experiments, for example, RNAseq or microarray profiling. Analytic pipelines that extract so-called signatures suffer from the "Dimensionality curse": the number of genes expressed exceeds the number of patients we can enroll in the study and use to train the discriminator algorithm. Hence, problems with the reproducibility of gene signatures are more common than not; when the algorithm is executed using a different training set, the resulting diagnostic signature may turn out to be completely different.

In this paper we propose an alternative novel approach which takes into account quantifiable expression levels of all genes assayed. In our analysis, the cumulative gene expression pattern of an individual patient is represented as a point in the multidimensional space formed by all gene expression profiles assayed in given system, where the clusters of "normal samples" and "affected samples" and defined. The degree of separation of the given sample from the space occupied by "normal samples" reflects the drift of the sample away from homeostasis in the course of development of the pathophysiological process that underly the disease. The outlined approach was validated using the publicly available glioma dataset deposited in Rembrandt and associated with survival data. Additionally, the applicability of the distance analysis to the classification of non-malignant sampled was tested using psoriatic lesions and non-lesional matched controls as a model.

**Keywords: **biomarkers; clustering; human diseases; RNA

## Introduction

The typical application of gene expression signatures for diagnosis and prediction of the course of disease is based upon an oversimplified understanding of the pathology. According to this model, there is a gene or a set of genes (say, a "gene expression program") that is "responsible" for a pathophysiological process within certain tissue, or a cell type, that manifests itself on an organismal level as a disease. If we see this gene over- or underexpressed, or observe a set of concerted changes in expression of a set of genes, we can diagnose the disease. We can further use the respective levels of the over- or underexpression to predict the course of the disease.

While many diseases are well described by this model, some--like many cancers-- are not. Often we deal with a system-wide changes of entire gene expression profile that involve many cellular pathways and networks, some changes are being related to the pathogenesis, and some are of compensatory nature [1]. In the case of system- wide changes, the sheer number of the genes to be examined prevents unambiguous determination of a group of genes (using a more technical language, a linear combination of their expression levels) suitable as a diagnostic signature for the given disease. The problem is not with the procedure *per se*, but with a typically limited number of already diagnosed patients we could biopsy and enter into the analysis as a *training data set*. As shown by simulations in [2], the development of the robust gene signature require enrollment of thousands patients, which is not feasible (see also [3] for detailed discussion).

In a nutshell, the problem is that we are trying to profile many different genes simultaneously. In a typical high-throughput experiment assessing transcriptome, proteome or metabolome, the number of available tissue samples is much smaller than the number of variables [3]. This leads to a high probability of spurious correlations. Indeed, even if the probability for one gene expression level to show a spurious correlation with the disease within the given data set is as small as 10^− ^^3^, an analysis of data streaming from this experiment with 4 × 10^4 ^genes will almost certainly produce many false positives.

This problem is especially prominent in the analysis of mRNA microarray or the RNAseq experiments. When different groups extract diagnostic signatures for the same disease, the resultant sets of genes often have negligible overlap. For example, the tests [4] and [5] for breast carcinoma, having 76 genes and 70 genes correspondingly, have only 3 genes in common. Even starting from the same data set, one can get different "predictive" panels with minimal overlap [6]. The difference in gene sets extracted using different training sets is not limited to individual genes. The pathways and networks that can be built using independently obtained gene expression signatures are also quite different [7]. These observations cast substantial doubt at the biological relevance of the diagnostic approach that relies on gene signatures.

Expression levels for individual genes and other variables quantified in high- throughput biological experiments are commonly thought of as dimensions of the space on which we are collecting information. Thus the problem outlined above is known as the "curse of dimensionality" [8]. Briefly, in highly dimensional models, the number of parameters (dimensions) *p *is substantially larger than the sample size *n*. This property of biological datasets makes the task of distinguishing the noise from the true biological signal quite challenging, and it becomes close to impossible to obtain consistent estimator procedures [9,10]. Hence there is a need to develop integrative approaches, capable of combining data from multiple high-throughput experiments to increase sample size [9,10] or statistically sound and robust techniques to reduce the data to the most informative features. As an example of the latter approach, we can try to transform the entire dataset into a limited set of clusters using hierarchical clustering [11]: starting from the definition of a distance between two tissue samples, we proceed by regrouping individual expression profiles to obtain a branched cluster tree. Unfortunately, hierarchical clustering produces plausibly looking trees even when random data points are entered [12]. Hence, an extensive data perturbation by resampling is required for the validation of the obtained clustering [13]. Moreover, unsupervised classification techniques are far from being robust, as the inclusion of a new patient typically modifies original clustering.

Another popular solution to the dimensionality curse is to use a supervised approach that relies either on the pre-selection of the feature-limiting steps or on pre-filtering the data by the strength of an association of each variable with clinical outcome, or associations between variables [14,15]. Unfortunately, a majority of biological data analysts try a variety of data processing techniques before arriving at the final one that seems to be suitable to the dataset in question. Therefore this kind of supervision is inherently biased.

In this paper we propose an alternative novel approach based on the "distances" in the multidimensional space of gene expression values. As a proof-of-principle, we show that this approach produces surprisingly good results in separation of normal and affected samples both for analysis of human malignancies and for chronic progressive conditions like psoriasis.

### Multidimensional distances and clustering

A result of an expression experiment for a given sample is a (very long) vector that may be represented as a point in a multidimensional space. If we introduce a distance in this space, we can use the standard clustering techniques [16] to classify the points.

There are several ways to define the distance between two points, $X=\left[x_{1},x_{2},...\right]$ and $Y=\left[y_{1},y_{2},...\right]$ (see the discussion in [16]). The nai've Euclidean distance is defined as

(1) $$D_{\text{Euclidian}}^{2}=\underset{i}{\sum }{\left(x_{i}-y_{i}\right)}^{2}$$

It takes all components of vectors equally and does not account for any correlations between them. We can normalize the sums in equation (1) on the average vector length

(2) $$M_{i}^{2}=\left\langle x_{i}^{2}\right\rangle$$

where the averaging denoted by angular brackets is taken over all vectors in the dataset. Then the scaled distance is defined as

(3) $$D_{\text{scaled}}^{2}=\underset{i}{\sum }\frac{{\left(x_{i}-y_{i}\right)}^{2}}{M_{i}^{2}}$$

In the gene expression experiments the absolute values of the vectors are not relevant since the expressions are arbitrarily normalized. Therefore, using a distance that does not take these values into account is justified. A correlation distance, or Pearson distance, is the one that has this property. It is defined as

(4) $$D_{\text{Pearson}}=1-\underset{i}{\sum }\frac{x_{i}y_{i}}{\sqrt{\sum_{i}x_{i}^{2}\sum_{j}y_{j}^{2}}}=1-\frac{X\cdot Y}{\left|X||Y\right|}=1-\text{cos}\left(∠\left(X,Y\right)\right)$$

If we have enough data to calculate covariance matrix S with the elements obtaining by the averaging over all vectors in the dataset their covariances:

(5) $$s_{ij}=\left\langle x_{i}y_{j}\right\rangle$$

them we can use Mahalanobis distance

(6) $$D_{\text{Mahalanobis}}^{2}={\left(X-Y\right)}^{T}S^{-1}\left(X-Y\right)$$

This distance takes into account all correlations in the data. The problem with it is that to calculate correlation matrix and its inverse, many data points, i.e. many patients, are required.

Therefore, in the calculations below we use, as a rule, Pearson distance (4). Note that since all components of |X| and |Y| are non-negative, this distance is always between 0 and 1.

## Methods

To test for the practical usefulness of the distance-based expression metrics we deployed the following strategy:

1 The datasets were selected from public MIAME-compliant GEO repository http://www.ncbi.nlm.nih.gov/geo/.

2 For each subset of the samples within data set (normal tissues, affected tissues, etc.) we calculated the coordinate for the center of the space defined by points of all the vectors as the simple arithmetic mean of all the samples in the subset.

3 For each point we calculated the distance to the centers of all subsets.

4 The distances to one center *r*_1 _vs. the distance to another center *r*_2 _were plotted.

In some datasets, three different subsets may be defined, instead of two. In these cases, each point was associated with three distances *r*_1_, *r*_2 _and *r*_3_, each plotted to the center of the corresponding subset. In these cases we used barycentric coordinates [17] in a equilateral triangle, with the distance *d_i _*from the vortex i proportional to

(7) $$d_{i}=\frac{r_{i}}{r_{1}+r_{2}+r_{3}}$$

Note that most programs dealing with barycentric coordinates (for example, the popular package [18]) use a different definition of the coordinate system, popular in the analysis of ternary mixtures. With this definition the distance to the vortex is proportional to *D_i _*= 1 − *d*_i_. The transformation between these two definitions is trivial:

(8) $$D_{i}=\frac{1}{2}\left(1-\frac{d_{i}}{\sum_{j=1}^{3}d_{j}}\right)$$

## Results

### Expression profiles of primary tumors and their metastases drift away from the homeostatic state

To test the hypothesis that the expression profiles of primary tumors and their metastases drift away from the healthy, homeostatic state, the RNAseq dataset with GEO Series accession number GSE46622 described in [19] was downloaded and reanalyzed. This dataset was generated using RNAseq profiling of matching normal, tumor and metastasis tissues from eight colorectal cancer patients. In the study, adaptor-clipped Illumina Genome Analyser IIx reads were mapped to the human genome version GRCh37 (hg19) using transcript models taken from Ensemble v64 with TopHat followed by determination of differential expression using the Cufflinks software bundle and the cuffdiff with upper quartile normalization [19].

**Figure 1:**
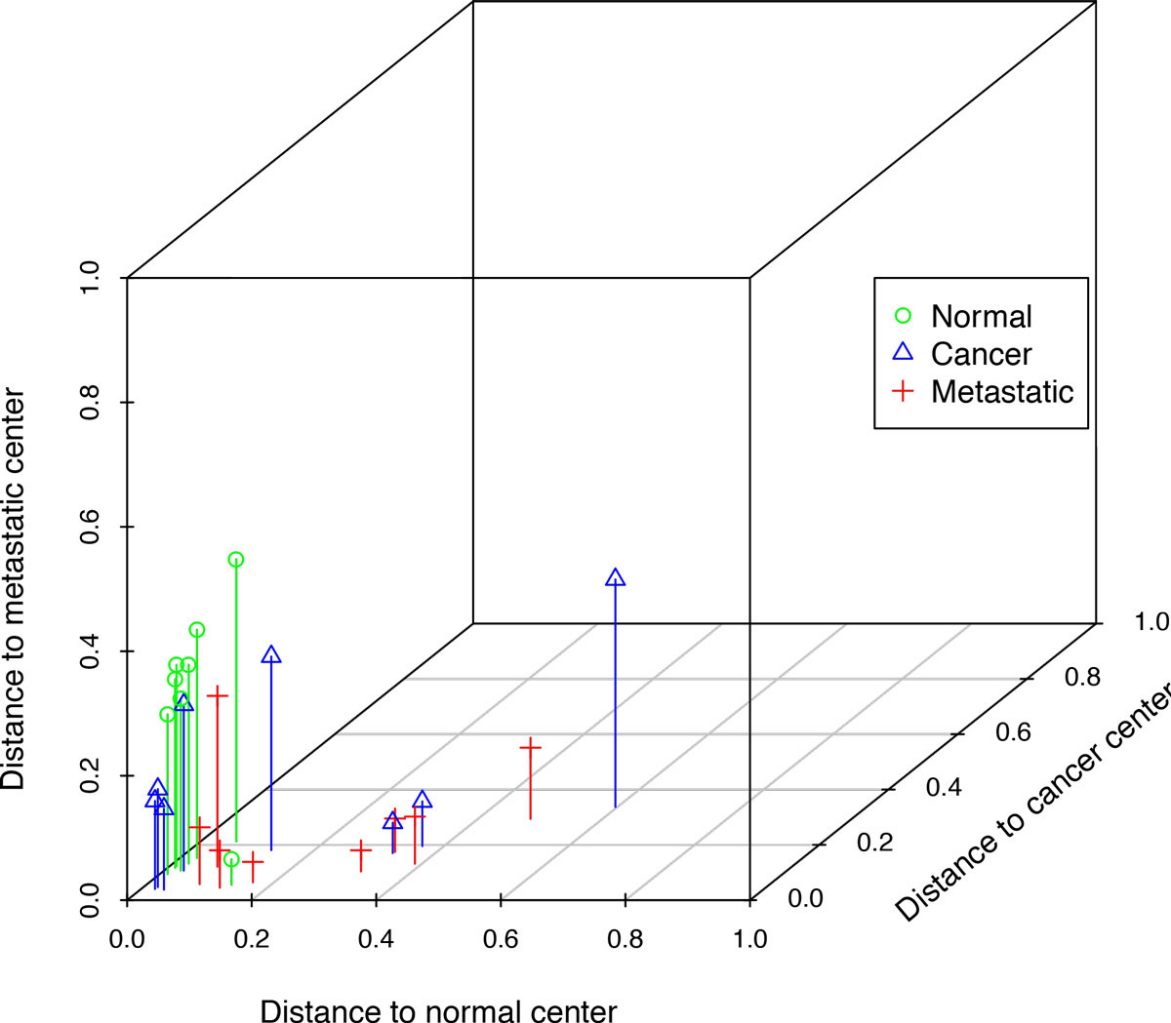
**Distances from cluster centers for colorectal cancer, 3D plot**. Each sample is represented as a point in the three-dimensional space defined by the distances to the normal, cancer, and metastatic center.

**Figure 2:**
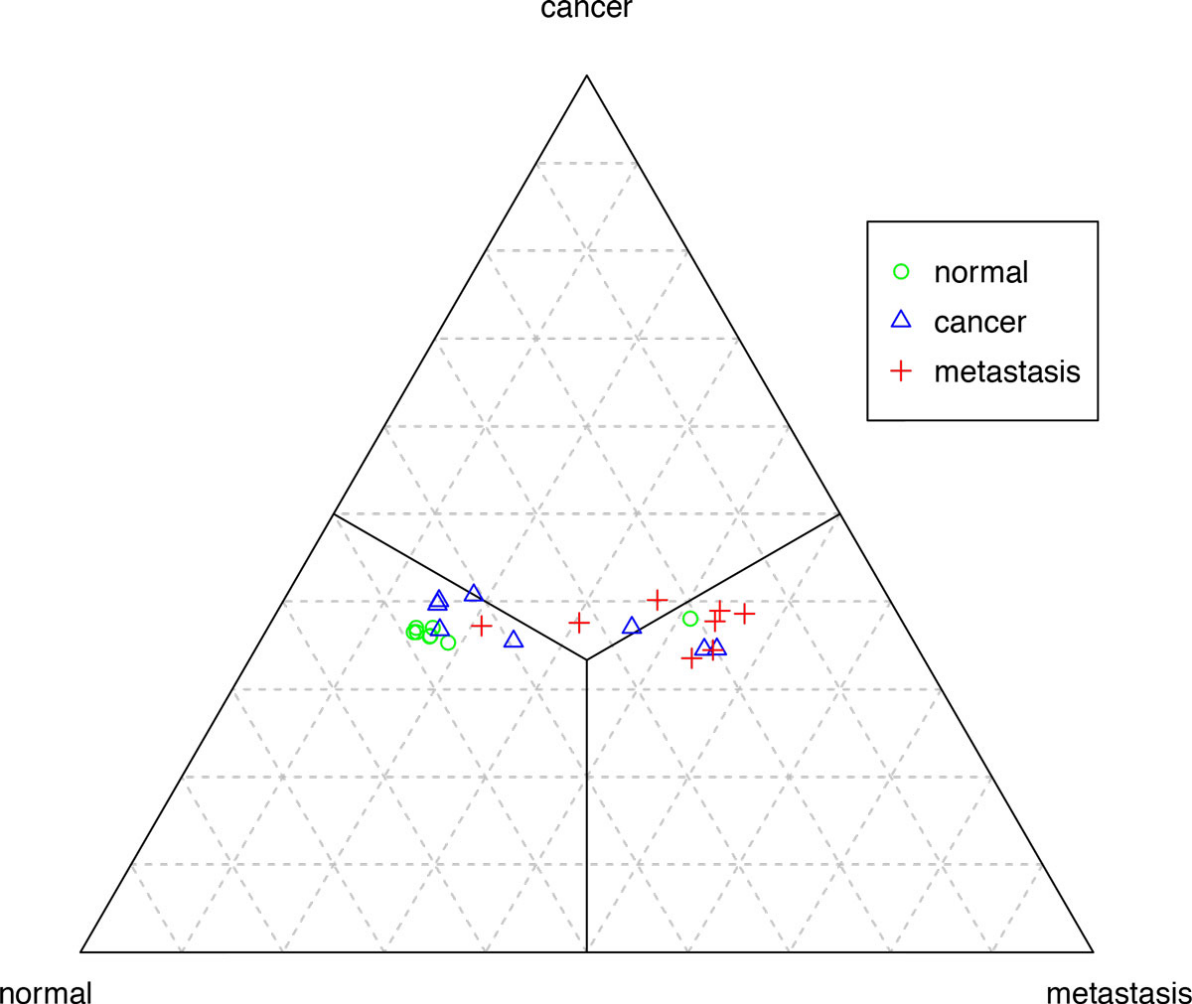
**Distances from cluster centers for colorectal cancer, barycentric plot**. **Plot of the distances in barycentric coordinates**.

Accordingly we had three different subsets and three different centers of clusters. These data may be presented either as the points within a 3d cube (Figure 1), or as barycentric diagram (Figure 2). Both diagrams show that normal samples are located relatively close to the normal center, while metastatic and cancer clusters are much less compact.

**Figure 3:**
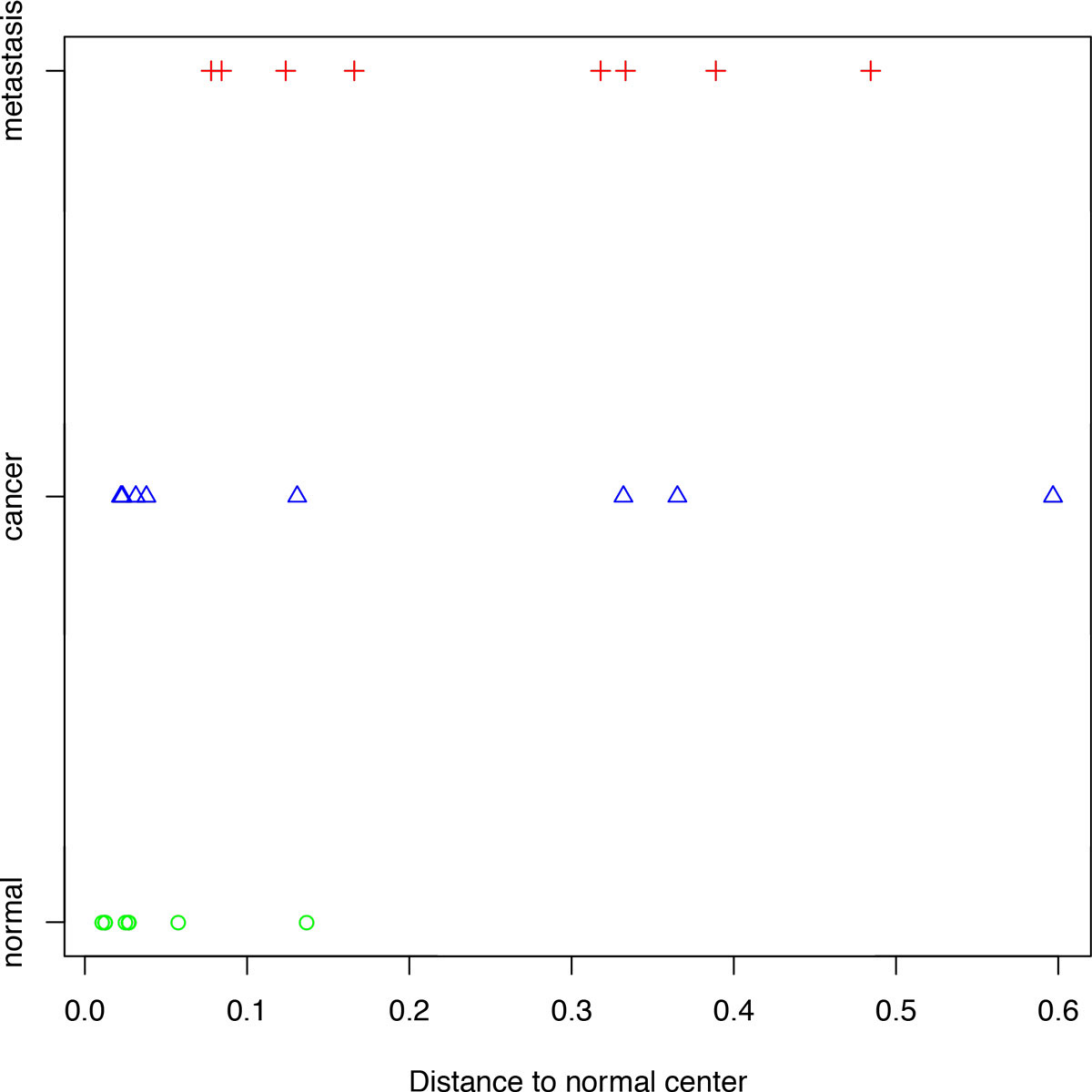
**Distances from the normal center for colorectal cancer**. Plot of the distance to normal center for colorectal cancer and different groups of samples.

In a practical sense, one may be interested in how far away given tumor sample departed from the center of the space occupied by normal samples. This approach allows one to draw a linear plot (Figure 3). Interestingly, not all normal samples are clustered tightly around the center of the normal space, at least one of them strays away into the space area that doubts its normal origin. This observation may be explained by the fact that in this particular dataset, all normal samples were derived from margins of the colon tumors, therefore, the sample in question may not be entirely normal. Of note, the distance-based spread in primary tumors was even large than that in metastasis derived from the same group of patients, supporting the "metastatic dormancy" theory posing that a disseminated tumor cell remains in a quiescent state at a remote organ for years before its reactivation in response to both an intrinsic program and a set of contextual cues [20]. Metastatic reactivation does not require the transcriptional profile be farther away from the normal center than that of respective primary tumor. However, the relative distance may be related to the total number of cell divisions passed from the onset of tumorigenesis; in the metastatic site that remained dormant for a long time, the number of cell divisions passed may be, in fact, lower than that in the site of the primary tumor.

### The drifting distances of tumor samples reflect the degree of their relative malignancy

To prove this point, we downloaded data represented in publicly available Repository for Molecular Brain Neoplasia Data (Rembrandt) http://caintegrator.nci.nih.gov/rembrandt/, which included data on 21 normal samples, 221 glioblastoma multiforme (GBMs), 145 astrocytomas, 66 oligodendrogliomas and 11 tumors of mixed origin. The raw gene expression CEL files from Affymetrix HGU133 Plus 2.0 arrays were normalized using the robust multi-array average (RMA) method [10] with default parameters [21].

**Figure 4:**
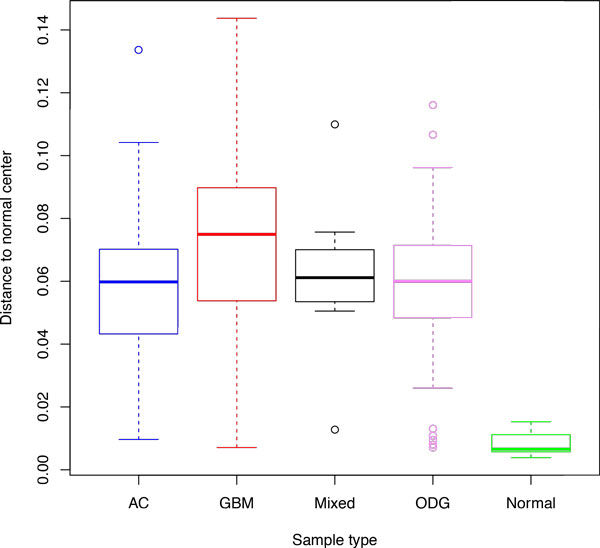
**Distances from the center of normal samples for multiple cancers, part I**. Boxes correspond to the second and third quartiles of the data.

**Figure 5:**
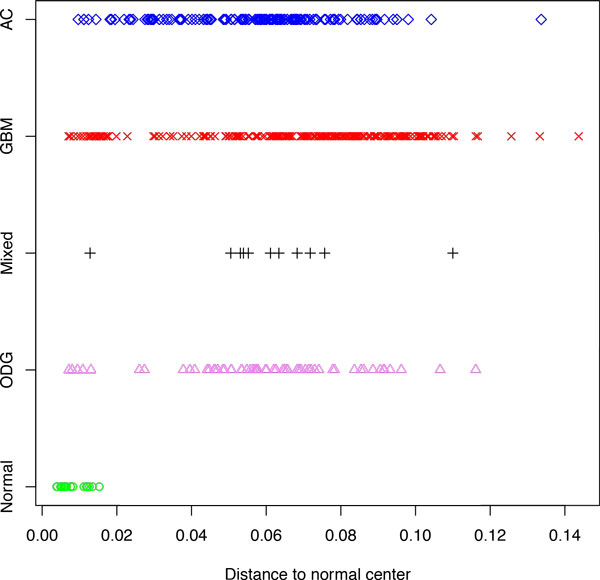
**Distances from the center of normal samples for multiple cancers, part II**. Points represent individual samples.

The plots reflecting the distances to the center of normal samples for all studied groups of samples are shown on Figures 4 and 5. Similarly to the the pattern observed using colorectal dataset normal samples were compacted close to the normal center, while the majority of tumor samples drifted away from norm. Glioblastoma multiforme samples were, on average, located further from the center of normal space than either astrocytomas (*p *= 3.1 × 10^−6^) or oligodendrogliomas *(p *= 0.0033). In both astrocytomas and oligodendrogliomas, the observed spreads of the distance values were quite large, possibly reflecting known heterogeneity of these tumors [22,23].

**Figure 6:**
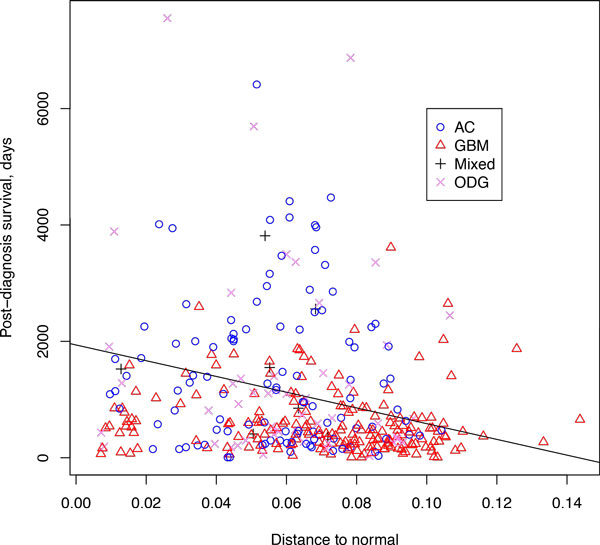
**Dependence of the post-diagnosis survival on the distance to then normal center for multiple cancer**. The line corresponds to the fit *y *= 1.94 × 10^3 ^- 1.35 × 10^4^*x*.

Interestingly, patients survival were found to be negatively correlated with the distance of the tumor expression profile to the center of normal samples: the farther was the expression profile from the homeostatic center defined by normal samples, the shorter was the survival length for the patient. The corresponding plot is shown on Figure 6 together with the linear fit. The fit results give *p *= 2 × 10 ^−9 ^and *R*^2 ^= 0.09. This means that the dependence is statistically significant, but the degree of the drift away from the center of the normal space explains only 9% of the survival prognosis, while the remaining 91% is explained by other factors, most likely, particular chromosomal rearrangements and mutations observed within the tumor along with tumor location and other known determinants of glioma prognosis [24].

### Distance analysis is applicable to classification of samples collected from patients with non-malignant chronic disease

**Figure 7:**
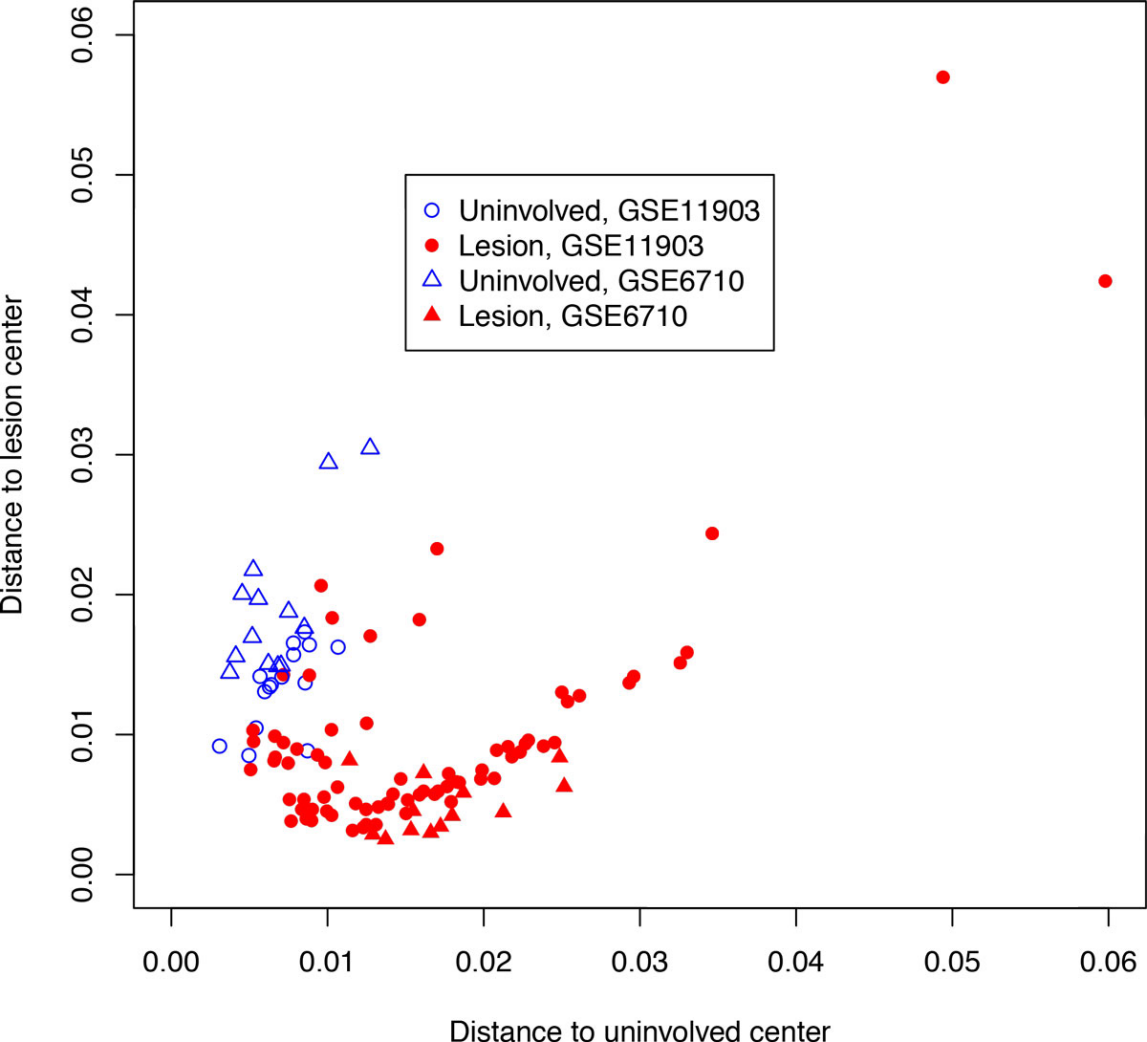
**Distances from the cluster centers for psoriasis data**. Unfilled blue symbols correspond to uninvolved skin, filled red symbols correspond to lesion skin.

**Figure 8:**
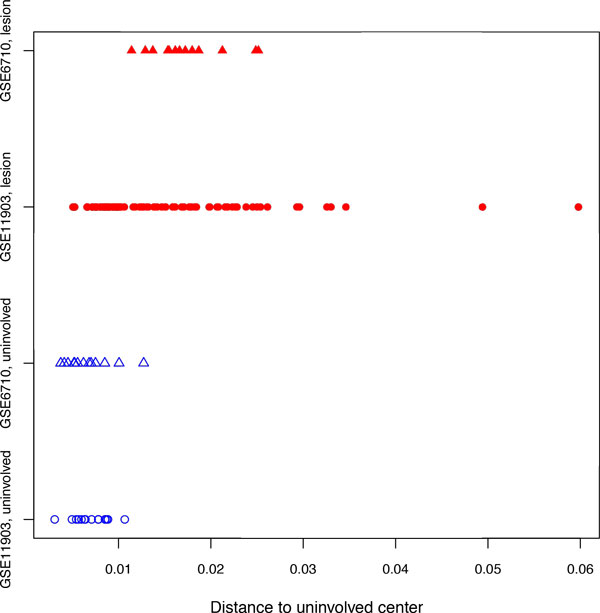
**Distances from the uninvolved center for psoriasis data**. Unfilled blue symbols correspond to uninvolved skin, filled red symbols correspond to lesion skin.

To illustrate an applicability of the gene expression distance analysis to the classification of samples collected from patients with non-malignant chronic disease, we selected psoriasis, an autoimmune disease mainly diagnosed through the visual inspection of the lesion skin by experienced dermatologists. To analyze the properties of gene expression profiles of lesional and non-lesional psoriatic skin samples from the same patient, two datasets were downloaded from GEO, GSE6710 [25] (*N *of paired samples = 13) and GSE11903 [26] (*N *of paired samples = 15). Both datasets were created using Affymetrix Human Genome U133A Array platform. As could be seen on Figures 7 and 8, even without cross-normalization, the distance metrics were able to cluster together gene expression profiles obtained using two independent non-lesional sample sets, while gene expression profiles of lesion samples were somewhat removed from the center of space occupied by non-lesional skin samples.

## Discussion

To date, the quantification of the diagnostic and prognostic biomarker molecules in the human serum and tissues, including cancer specimens, remains the primary means of enhancing the clinician's ability to diagnose the chronic condition. Importantly, with innumerable molecular markers in development, the discovery of novel standalone biomarkers with acceptable sensitivity and specificity is an extremely rare event.

Here we challenge the biomarker paradigm by developing a distance measure that places each tissue sample by its entire tissue-wide transcriptome profiles within the space occupied by similarly obtained profiles of the samples collected from the same individual or from individuals that do not have given chronic condition. We hypothesize that as farther away individual sample drifts from its homeostatic state defined as center of the space occupied (defined) by a large number of reference (normal) samples, as farther away the respective tissue will be from the well maintained, healthy state. In our study, we used publicly available datasets, to develop easily interpretable, composite measure that capable of integrating high-throughput transcriptome profiles into comprehensive, holistic metric describing the molecular homeostasis within given sample.

The comprehensive distance measures account for the intrinsic heterogeneity of human tumors that plagues hight-throughput studies involving this type of the biological material [27] and even for a heterogeneity of the cell types that comprise given tissue [28]. In particular, the composite biomarker metric that we call a distance metric, was validated using well-known Rembrandt glioma dataset associated with survival outcomes.

Importantly, proposed composite biomarker may be suitable for a dynamic description of patients' condition. This novel concept allows one to depart from the classical two-bin prediction model (e.g. "bad prognosis/good prognosis") as it produces a continuous prognosis model, where each sample is located in the neighborhood of other samples analyzed post-hoc and associated with known survival. For each sample, this concept quantitatively describes the degree of "the drift" from the standardized phenotype that will reflect the departure of the body from homeostasis. In the concepts, the effects of each personalized intervention could be evaluated by comparing the distance metrics for samples collected before the treatment and at multiple time-points within the interventional treatment course.

If proven valid, this concept might be developed into a novel type of integrative tests for the monitoring of the disease progress and the prediction of disease outcomes. The proposed distance analysis has a potential to become versatile in its application as it is equally attributable to gene expression profiles collected both by microarrays and by RNA-seq platforms, as well as, possibly, to proteome and metabolome profiles.

There is no doubt that proposed computational approach requires further development and optimization, in particular, other types of correlation-based metrics have to be tested for various kinds of multiparametric datasets that comprise simultaneously measured analytes. Future studies should include an analysis of longitudinal experiments that involve either various time points in course of the therapeutic treatment that ultimately results in the normalization of the pathological condition, or gradual processes detrimental to experimental system, for example, a development of insulin resistance or an ageing.

## Conclusion

The distance analysis of molecular portraits is robust and versatile in its application as it is equally attributable to gene expression profiles collected by microarrays and by RNA-seq. The distance-based continuous predictive models depart from the classical two-bin prediction model (e.g. "bad prognosis/good prognosis") by placing each sample in the neighborhood of other samples analyzed post-hoc and associated with known survival.

## Competing interests

The authors declare that they have no competing interests.

## Authors' contributions

BV drafted the manuscript, provided insights concerning dimensionality curse and mathematical advice in the course of this study. AB conceived this study and developed the concept of distance metrics. LW and TC analyzed both cancer datasets and contributed to the draft of the manuscript. SB analyzed psoriasis dataset and contributed to the development of the concept of distance metrics.

## Declarations

The publication charges were covered by Vavilov Institute of General Genetics RA.

This article has been published as part of *BMC Genomics *Volume 15 Supplement 12, 2014: Selected articles from the IX International Conference on the Bioinformatics of Genome Regulation and Structure\Systems Biology (BGRS\SB-2014): Genomics. The full contents of the supplement are available online at http://www.biomedcentral.com/bmcgenomics/supplements/15/S12.
